# Transition of Eocene Whales from Land to Sea: Evidence from Bone Microstructure

**DOI:** 10.1371/journal.pone.0118409

**Published:** 2015-02-25

**Authors:** Alexandra Houssaye, Paul Tafforeau, Christian de Muizon, Philip D. Gingerich

**Affiliations:** 1 UMR 7179 CNRS/Muséum National d’Histoire Naturelle, Département Ecologie et Gestion de la Biodiversité, Paris, France; 2 Steinmann Institut für Geologie, Paläontologie und Mineralogie, Universität Bonn, Bonn, Germany; 3 European Synchrotron Radiation Facility, Grenoble, France; 4 Sorbonne Universités, CR2P—CNRS, MNHN, UPMC-Paris 6, Département Histoire de la Terre, Muséum National d’Histoire Naturelle, Paris, France; 5 Department of Earth and Environmental Sciences and Museum of Paleontology, University of Michigan, Ann Arbor, Michigan, United States of America; New York Institute of Technology College of Osteopathic Medicine, UNITED STATES

## Abstract

Cetacea are secondarily aquatic amniotes that underwent their land-to-sea transition during the Eocene. Primitive forms, called archaeocetes, include five families with distinct degrees of adaptation to an aquatic life, swimming mode and abilities that remain difficult to estimate. The lifestyle of early cetaceans is investigated by analysis of microanatomical features in postcranial elements of archaeocetes. We document the internal structure of long bones, ribs and vertebrae in fifteen specimens belonging to the three more derived archaeocete families — Remingtonocetidae, Protocetidae, and Basilosauridae — using microtomography and virtual thin-sectioning. This enables us to discuss the osseous specializations observed in these taxa and to comment on their possible swimming behavior. All these taxa display bone mass increase (BMI) in their ribs, which lack an open medullary cavity, and in their femora, whereas their vertebrae are essentially spongious. Humeri and femora show opposite trends in microanatomical specialization in the progressive independence of cetaceans from a terrestrial environment. Humeri change from very compact to spongious, which is in accordance with the progressive loss of propulsive role for the forelimbs, which were used instead for steering and stabilizing. Conversely, hind-limbs in basilosaurids became strongly reduced with no involvement in locomotion but display strong osteosclerosis in the femora. Our study confirms that Remingtonocetidae and Protocetidae were almost exclusively aquatic in locomotion for the taxa sampled, which probably were shallow water suspended swimmers. Basilosaurids display osseous specializations similar to those of modern cetaceans and are considered more active open-sea swimmers. This study highlights the strong need for homologous sections in comparative microanatomical studies, and the importance of combining information from several bones of the same taxon for improved functional interpretation.

## Introduction

Many amniote groups (e.g. sauropterygians, squamates, cetaceans, sirenians, pinnipeds) made the evolutionary transition from a fully terrestrial to a semi- to fully aquatic life. This required major morphological and physiological changes that are best developed in the most specialized aquatic forms, like extant cetaceans and sirenians, which now live totally independent of the terrestrial environment. Several lineages are known with transitional fossil forms, but it remains difficult to determine both their degree of physiological adaptation to an aquatic milieu and their locomotor ability in water. Better knowledge of these intermediate forms is essential for understanding the process of secondary adaptation to life in water.

**Fig 1:**
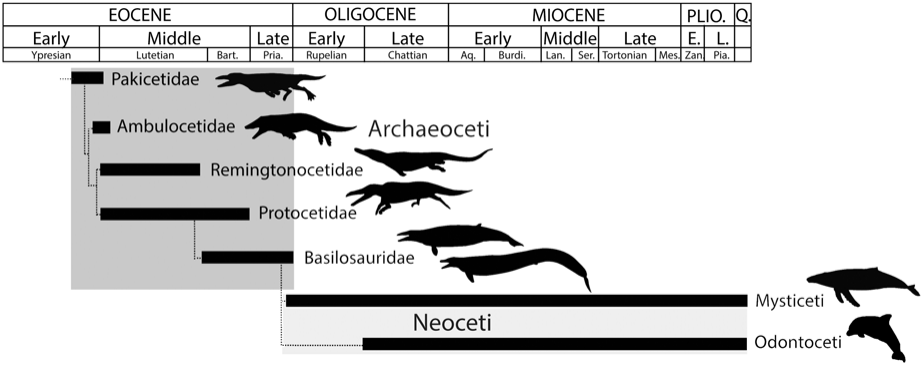
Phylogenetic relationships of early cetaceans showing the temporal ranges and general relationships of Pakicetidae, Ambulocetidae, Remingtonocetidae, Protocetidae, and Basilosauridae discussed here. Modified from [75,76].

Here we focus on the transition of cetaceans from land to sea. Cetaceans arose in the early Eocene (about 50 Myr ago), when the earliest fossils are known in Indo-Pakistan. ‘Archaic’ or ‘primitive’ cetaceans, called archaeocetes, include five families illustrating various modes of adaptation to an aquatic life (Fig. 1). The degree of aquatic adaptation and swimming modes of these taxa are debated (e.g. [1–11]). Here we address the lifestyle of early cetaceans by analysis of microanatomical features in postcranial elements of the three more derived archaeocete families, Remingtonocetidae, Protocetidae, and Basilosauridae, extending research by Buffrénil et al. [12], Madar [13,14] and Gray et al. [15].

### (a) Remingtonocetidae

Early middle Eocene Remingtonocetidae have skeletons indicating that they were long-bodied, with a long cranial rostrum, short limbs, fused sacral vertebrae, and a powerful tail [16,17]. They are considered an early aquatic radiation with distinct specializations [6,11], and are sometimes interpreted as amphibious with an otter-like or gavial-like mode of swimming [6,18]. Bebej et al. [19] showed that terrestrial abilities were limited in remingtonocetids, and that propulsion during swimming was powered by the hindlimbs rather than undulation of the lumbar region. This is consistent with their sense organs being poorly compatible with terrestrial locomotion (small eyes, small semicircular canals; [20]). Both sedimentological and isotopic evidence suggests that remingtonocetids lived in coastal marine environments [6,10,21]. The specimens of *Remingtonocetus domandaensis* Gingerich et al., [22] that we analyze here came from the early middle Eocene (middle Lutetian) of Pakistan.

### (b) Protocetidae

Middle Eocene Protocetidae are a parallel radiation of early cetaceans evolving independently of Remingtonocetidae. Protocetids are found in Indo-Pakistan [2,16,22–24], but also in North Africa [25], West Africa [26], North America [27–29], and South America [30]. Protocetids were the first cetaceans to disperse widely through the world’s oceans.

Kellogg ([31], p. 277) regarded *Protocetus* as being “far advanced” in the transition to life in water, and “well adapted for a pelagic life.” This was partially confirmed when more complete protocetid skeletons were found [2,22,24,27,32]. Protocetids have a short lumbar region of the vertebral column, short ilium of the pelvis, and short femur, combined with relatively long manual and pedal phalanges. However, retention of well developed and powerful hind limbs connected to the vertebral column is an indication that protocetids were not yet fully aquatic. The characteristics of protocetids, taken together, indicate foot-powered swimming in a relatively aquatic mammal [7]. The pedal phalanges of protocetids are long and delicate relative to the size of the animal. While protocetids could still come out on land to give birth [24], they could not have moved far from a shoreline.

Protocetids have small semicircular canals in accordance with their limited terrestrial locomotion [33]. Early protocetids have a true pelvis with the innominates attached to a sacrum of 3 or 4 co-ossified vertebrae and functional hind limbs well articulated to the innominate. This arrangement provided the stable platform required for foot-powered swimming. Although the attachment of the innominates to a solid sacrum has been reduced (e.g. in *Natchitochia*, [34]) or possibly even lost in later members of the family (*Georgiacetus*, [27]), no protocetids are known to have been fully aquatic like later basilosaurids.

Here we analyse specimens of *Rodhocetus kasranii* Gingerich et al. [32], *Qaisracetus arifi* Gingerich et al. [22], and *Maiacetus inuus* Gingerich et al. [24], all from the early middle Eocene (Lutetian) of Pakistan.

### (c) Basilosauridae

Middle and late Eocene Basilosauridae are morphologically similar to modern cetaceans, with forelimbs modified into flippers retaining a mobile elbow, reduced hind limbs, and a powerful vertebral column with a tail fluke adapted for undulatory or oscillatory tail-powered swimming [11,35]. Basilosaurids had reduced hind limbs articulated to a pelvis lacking any bony connection to the vertebral column, and were undoubtedly fully aquatic. From the known fossil record, they were also fully marine. Basilosaurids like *Dorudon* and *Cynthiacetus* had body proportions close to those of recent dolphins or porpoises, but *Basilosaurus* had an exceptionally long body and tail, differing greatly from the other two genera in being more serpentine. *Basilosaurus* had a tail fluke, but the tail was probably not the only source of propulsion. *Basilosaurus* probably swam by undulation of the whole body (an anguilliform swimming mode), and Gingerich [7] even suggested that the propulsion may have included lateral as well as dorsoventral undulation.

Here we analyze specimens of *Basilosaurus isis* Beadnell in Andrews [36] and *Dorudon atrox* Andrews [37] from the late Eocene of Egypt, and of *Cynthiacetus peruvianus* Martínez-Cáceres & Muizon [38] from the late Eocene to early Oligocene of Peru.

### (d) Bone microanatomical features

Microanatomical features of bone include its internal structure and organization. These reflect and record the biomechanical response of bone as a living tissue to the stress and strain of organisms during life. Stress and strain are themselves a strong ecological signal (e.g. [39–43]).

Two alternative microanatomical specializations are found in virtually all strongly or exclusively aquatic amniotes that forage below the water surface [44]. These specializations of bone architecture involve either an increase in bone mass or the development of a spongy inner organization and are related to swimming ability through the control of buoyancy (see [45,46]).

Bone mass increase (BMI) is a specialization found in various groups of slow and relatively inactive, but essentially or even exclusively aquatic, subsurface swimmers like sirenians and various aquatic fossil reptiles (see [45] for a review). The latter display compact bone organization (osteosclerosis), which makes the bones brittle, with possible additional cortical bone deposits (pachyostosis). BMI by itself confers hydrostatic regulation of buoyancy and body trim.

A spongious inner organization is found in highly aquatic active swimmers (modern cetaceans, derived mosasaurs, ichthyosaurs, plesiosaurs). The latter display a spongy bone organization, with much reduced compact bone and a tight network of osseous trabeculae oriented in the direction of maximal stress, probably associated with a more even distribution of forces during active locomotion, in order to prevent breakage in a milieu of reduced gravity (see [46–48]). Osteoporosis requires hydrodynamic regulation of buoyancy and body trim.

Neither of these divergent specializations is considered compatible with terrestrial locomotion and neither is found with any frequency in terrestrial taxa [45].

Very little microanatomical information is available for archaeocete whales spanning the transition from land to sea. The relative distribution of compact and spongious bone has been reconstructed hypothetically for various archaeocete long bones based on radiographs reflecting essentially density differences [13]. However, bone compactness alone is not as good an indicator of behavior and ecology as bone compactness combined with the internal organization of bone (see [49]). Rib sections have been described in archaeocetes [12,15], as well as some fracture sections of long bones of pakicetids [14,50]. Buffrénil et al.’s [12] and Gray et al.’s [15] studies are based on broken rib fragments, whose position along the vertebral column or within a rib could not be specified, meaning that their observations have to be interpreted with caution.

Here we document much more of the internal structure of bone from various parts of the skeleton in archaeocete specimens belonging to three of the five known families. This enables a more substantial discussion of skeletal specialization observed in these taxa and offers greater constraint when discussing behavioral and ecological implications.

## Materials and Methods

We are thankful to C. Sagne and S. Sanchez for the loan and transport of the *Cynthiacetus* material. We thank the ESRF (Grenoble, France) and Steinmann Institut (University of Bonn, Germany) for providing beamtime and support, the ESRF in the framework of the proposal EC-774 on the beamline ID17.

### (a) Materials

We focused our study on archaeocetes from the three more derived archaeocete families—Remingtonocetidae, Protocetidae, Basilosauridae (see Table 1)—illustrating a wide spectrum of the diversity of this group after its earliest stages.

**Table 1:** List of material analyzed in this study.

Family	Species	Coll. number	B	BN	Vox. S
Remingtonocetidae	*Remingtonocetus domandaensis*	GSP-UM 3225	V (T10)		64.7*
			R	1	39.8*
				2	39.1*
				3	34.9*
		GSP-UM 3054	F		66.8*
					48.9*
Protocetidae	*Rodhocetus kasranii*	GSP-UM 3012	V (T6)		83.6*
			R	1	68.0*
				2	76.1*
				3	52.6*
				4	54.3*
			F		78.7*
					42.4*
	*Maiacetus inuus*	GSP-UM 3551	V (T4)		45.7#
			H		45.7#
			Ra		45.7#
			U		45.7#
			F		45.7#
			T		45.7#
	*Qaisracetus arifi*	GSP-UM 3410 Proximal part	V (T9)		86.0*
			R		43.2*
		GSP-UM 3323	V (T4)		91.1*
		GSP-UM 3318 Distal half	H		77.7*
					58.3*
*Basilosauridae*	*Dorudon atrox*	UM 101222 WH-224	V (T6)		45.7#
			R	1	55.9*
				2	75.5*
				3	65.2*
				4	42.6*
				5	49.1*
			H		45.7#
			Ra		45.7#
			U		45.7#
		UM 97506 (WH-072) Proximal half	F		45.1*
	*Basilosaurus cetoides*	USNM 510831a	V		-
		USNM 510831b	V		-
	Basilosaurus isis	UM 94803 (WH-009)	H		169.0*
		WH-074	R	1	63.2*
				2	74.6*
				3	100.7*
		UM 97527 (WH-152)	F		72.8*
		UM 93231 (WH-132)	F		86.6*
	*Cynthiacetus peruvianus*	MNHN.F.PRU 10	V (T7)		45.7#
		MNHN.F.PRU 10	H		45.7#

Abbreviations: B–bone, F–femur, H–humerus, R–rib, Ra–radius, T–tibia, U–ulna, V–vertebra, and Vox. S–voxel size. BN is the block number, with numbering increasing proximodistally.

*: scanned at the Steinmann Institut (Bonn, Germany).

# scanned at the ESRF (Grenoble, France);—not scanned.

GSP-UM: Geological Survey of Pakistan-University of Michigan, specimens archived in Quetta, Pakistan; MNHN: Muséum national d’Histoire naturelle, Paris, France; UM: University of Michigan Museum of Paleontology, USA.

We analyzed long bones from the stylopod (humerus and femur) and from the zeugopod (radius, ulna, tibia). We also analyzed ribs and thoracic vertebrae (except for *Basilosaurus*, where we analyzed lumbar or anterior caudal vertebrae). Our choice was supported by suggestions from previous studies that proximal limb bones should provide a stronger behavioral and ecological signal than more distal ones [51], and that vertebrae and ribs located above the lungs generally play an important role in buoyancy control [45]. All specimens sampled are true adults, based on tooth eruption and/or epiphyseal fusion, except the holotype of *Cynthiacetus peruvianus* that is proposed to be a young adult [38].

### (b) Methods

The fossils analyzed here are rare and parts of exceptionally complete skeletons, meaning that destructive sampling was precluded. No permits were required for the described study. Osteological cross sections were obtained from microscale computed tomography (CT), allowing non-destructive imaging of the three-dimensional outer and inner structure of the samples. Both conventional and synchrotron X-ray micro-CT (see Table 1) were used: (1) high-resolution computed tomography (GEphoenix∣X-ray v∣tome∣xs 240) was used at the Steinmann-Institut, University of Bonn (Germany), with reconstructions performed using datox/res software; and (2) third generation synchrotron propagation phase-contrast micro-CT [52] at the European Synchrotron Radiation Facility (ESRF, Grenoble, France), on beamline ID 17. The scans were performed with 5 meters of propagation, using a detector giving an isopetric voxel size of 45.71 µm. The energy was set at 100 keV using a double Laue Laue Si 111 bendable crystal monochromator. Most of the specimens being very large and dense, a specific protocol to optimize the X-ray transmission profile through the sample was used [53,54], allowing high quality scans despite transmission lower than 1%. Reconstructions were performed using a filtered back-projection algorithm with ESRF PyHST software.

Complete bone shafts could be scanned in conventional microtomography but only a short mid-diaphyseal section was scanned via synchrotron microtomography due to limited access to beam time. Image segmentation and visualization of resulting data were performed using Avizo 6.3. (VSG, Burlington MA, USA) and VGStudioMax 2.0. and 2.2. (Volume Graphics Inc., Heidelberg, Germany).

Virtual thin-sections were made in cross-sectional planes of interest that serve as a reference for comparative studies. These were longitudinal and mid-diaphyseal transverse sections for long bones, mid-sagittal and neutral transverse sections for vertebrae (see [55]). Following initial analyses, additional transverse virtual sections were made for long bones (see below). Rib transverse and longitudinal virtual thin sections were made at different positions along the bone (with the number of sections depending on rib preservation). Two lumbar or anterior caudal vertebrae of *Basilosaurus* were sectioned along their mid-sagittal and mid-transverse planes respectively. Finally, for long bone and rib sections, a compactness index (CI) was calculated representing the cross-sectional area occupied by bone as a percentage of total cross-sectional area.

The histological terminology is based primarily on Francillon-Vieillot et al. [56].

## Results

### (a) Remingtonocetus

Rib

There is no open medullary cavity. The rib displays a spongious organization. Cavities are fairly large in the medullary area and smaller in the cortex, which is much more compact. The latter displays some circumferential lines, which probably correspond to lines of arrested growth (LAGs—illustrating the cyclical growth), indicating that it is only feebly remodelled (as these primary structures are not remodelled). The relative thickness of the cortex decreases distally, while the tightness of the spongiosa slightly increases, trabeculae and intertrabecular spaces becoming slightly thinner and smaller respectively. Compactness is fairly high in the proximal part of the rib (CI~80) but lower in the distal one (CI~ 67).

Vertebra

The vertebra is spongious but a layer of compact cortex surrounds the bone periphery and the neural canal. Trabeculae are sagittally oriented in the longitudinal section. The spongiosa is much looser in the periosteal than in the enchondral territory.

Femur

**Fig 2:**
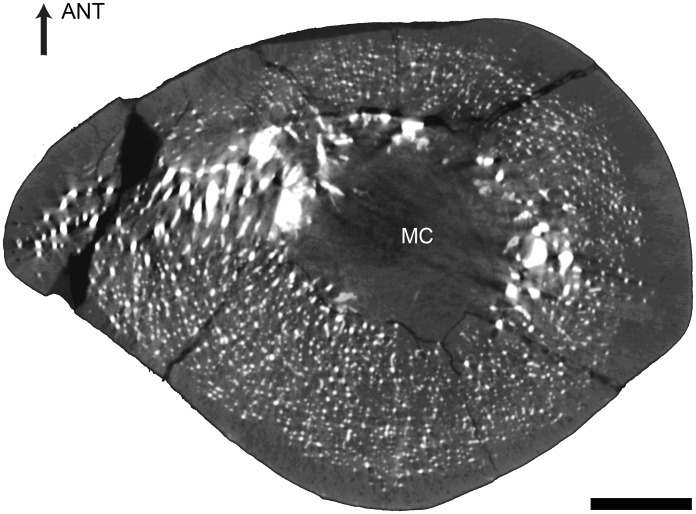
Left femur of *Remingtonocetus domandaensis*, GSP-UM 3054, virtual diaphyseal cross section. Section located just below the lesser trochanter, about one third of the length of the bone from the proximal end. MC: medullary cavity. The contrast between bone and the infilling sediment shows that the MC is open. Scale bar equals 5 mm.

The femur displays a thick cortex rather compact in its inner part and extremely compact in its periphery, and an off-center open medullary cavity (Fig. 2). The compactness index is rather high (82) proximal to the mid-diaphysis (Fig. 2).

### (b) Rodhocetus

Rib

**Fig 3:**
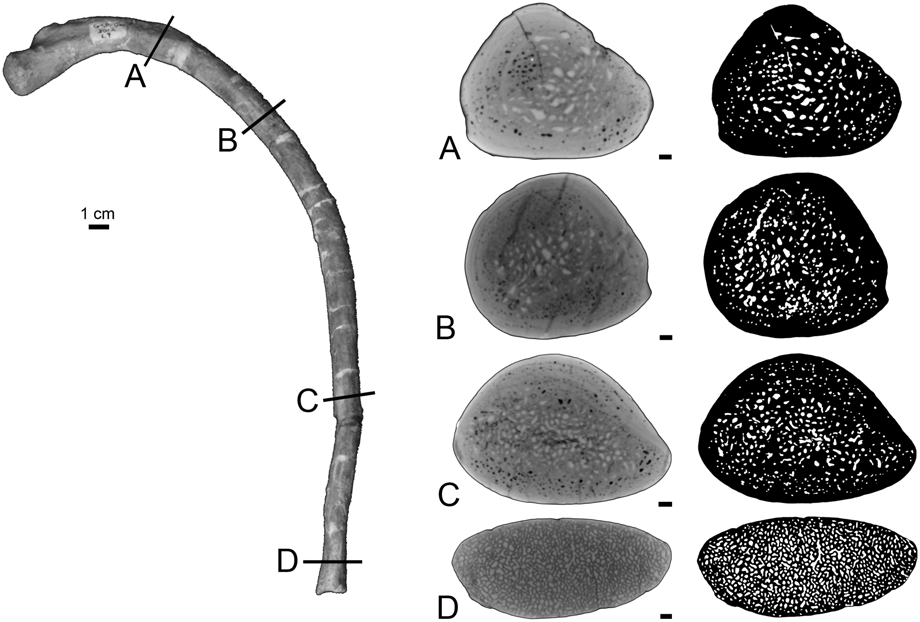
Left rib 9 of *Rodhocetus kasranii* GSP-UM 3012, in anterior view. A-D, virtual transverse sections (left) and corresponding binary images (right; in black: bone; in white: cavities) following the positions labelled on the rib. Scale bars equal 1 mm.

The rib lacks any open medullary cavity (Fig. 3). It displays a spongious organization but is highly compact. The first two thirds of the rib show a distinct surrounding layer of more compact periosteal bone, cavities being more numerous and larger in the medullary area (Fig. 3A-C). Some LAGs are observed in the cortex, which appears thus poorly remodelled. Compactness is high in the first two thirds of the rib (89.4<CI<91.8 in the sections analyzed). In the distal part of the rib the spongiosa becomes much tighter and occupies almost the whole section (Fig. 3D), so that compactness decreases (CI = 73.9).

Vertebra

**Fig 4:**
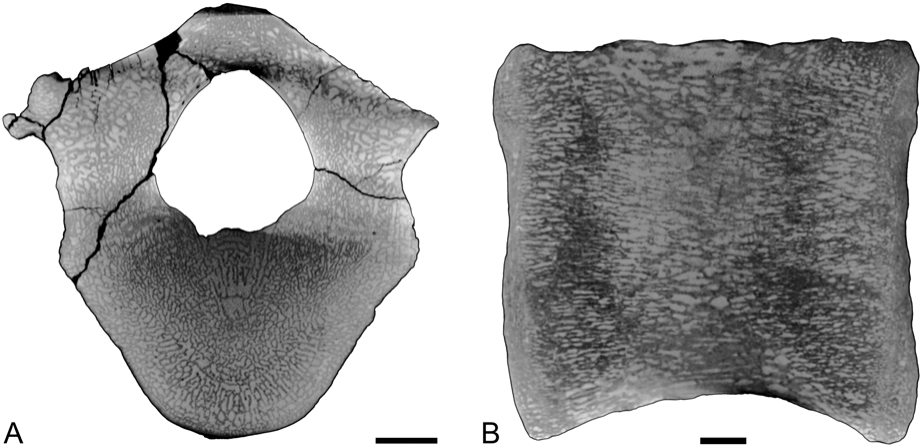
Vertebral virtual sections. A, *Rodhocetus kasranii* GSP-UM 3012, transverse virtual section of thoracic vertebra T6. B, *Qaisracetus arifi* GSP-UM 3410, mid-sagittal section of centrum of thoracic vertebra T9. Scale bars equal: A, 10 mm; B, 5 mm.

The vertebra is cancellous (Fig. 4). It is similar to that of *Remingtonocetus*, except in the absence of layers of compact bone. The transverse section illustrates a circumferential orientation of the trabeculae in the outer part of the centrum.

Femur

**Fig 5:**
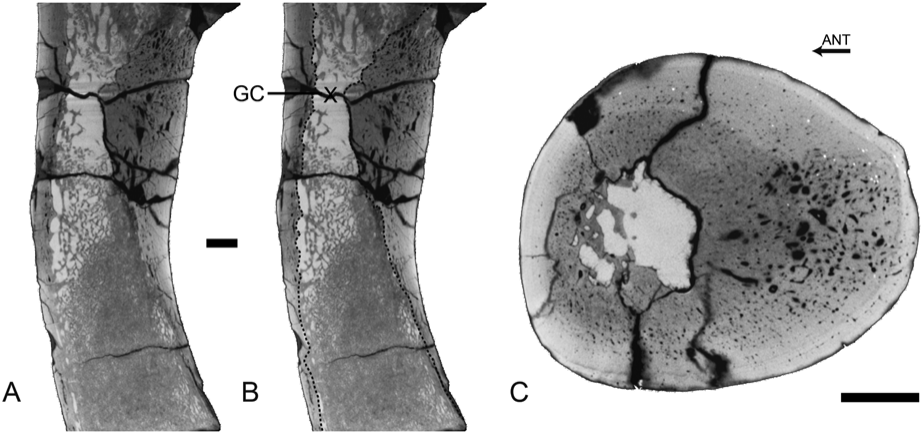
Left femur of *Rodhocetus kasranii* GSP-UM 3012. A-B, partial longitudinal section in lateral view; proximal is at the top. Limits of the compact cortex (dotted lines), as well as the position of the growth center (GC), are indicated on B; C, transverse section cutting the growth center. Scale bars equal 5mm. Cavities are either filled by sediment (light grey) or by epoxy (black) resulting from bone preparation.

The femur of *Rodhocetus* resembles that of *Remingtonocetus*, although it is more compact. The longitudinal section shows that the inner organization of the bone changes markedly along the diaphysis (Fig. 5A). The growth center, i.e., the point where growth originated, corresponds to the point of the transverse section displaying the thicker remains of the original cones of primary periosteal bone where the cones of endochondral and periosteal origin intersect (Fig. 5B). The growth center is usually located close to the mid-diaphysis, but here it appears clearly proximal (Fig. 5A). Around this point, the bone is strongly compact (CI = 83.6 and 87.4 on two different sections). The open medullary cavity is clearly off-center and surrounded by a cortex displaying numerous fairly small cavities, although they are larger posteriorly (Fig. 5C). Proximal and distal to the open medullary cavity, the micro-organization changes rapidly to more spongious bone.

### (c) Maiacetus

Most *Maiacetus* long bones show cracks or slight distortion so that compactness indices (measured at mid-shaft) are difficult to calculate and can only be estimates.

Vertebra

The vertebral microanatomical features are similar to those observed in the vertebra of *Rodhocetus*.

Humerus

**Fig 6:**
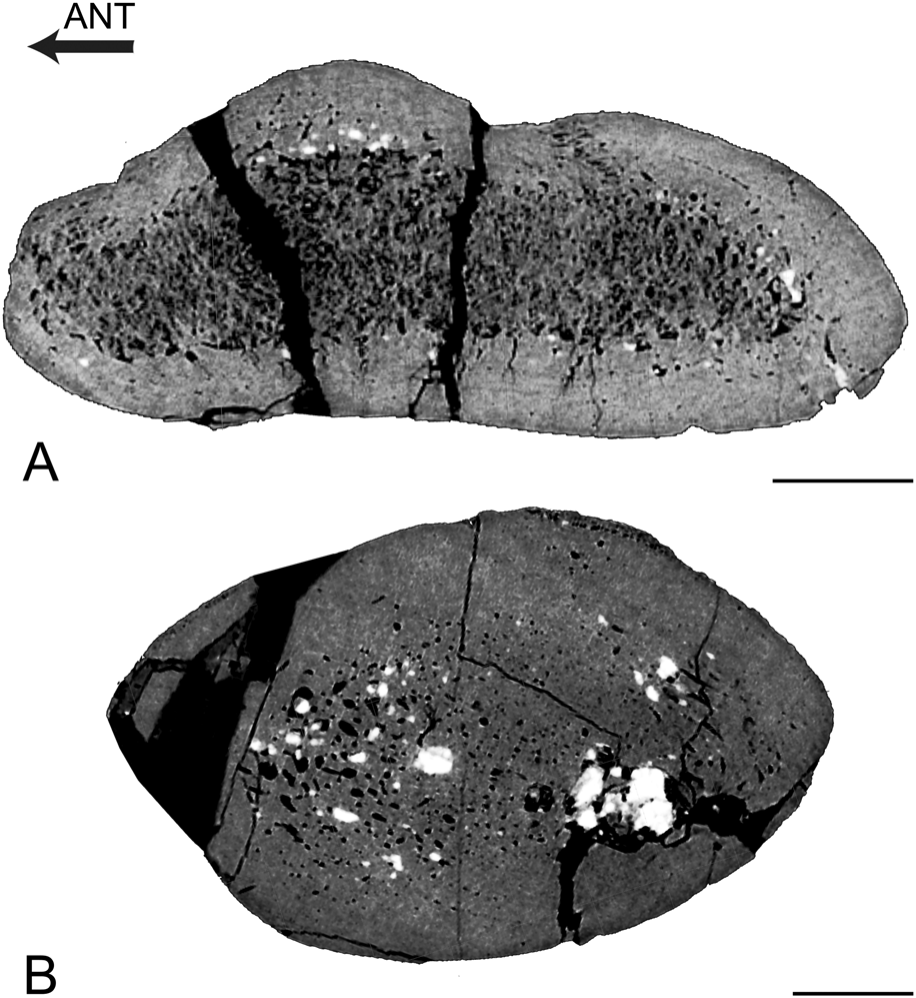
*Maiacetus inuus* GSP-UM 3551. Virtual transverse sections. A- Right humerus; B- Left femur. Scale bars equal 5mm.

The humerus of *Maiacetus* has, at mid-shaft, a rather thick layer of compact cortex surrounding an entirely spongious medullary area, with the contrast between the two being very sharp (Fig. 6A). Compactness is estimated at around 69%.

Radius and ulna

These bones display a microanatomical organization similar to that of the humerus. However, the layer of compact cortex at mid-diaphysis is proportionally thicker in the radius, so that compactness is higher in the latter than in the ulna (about 73% in the radius versus about 63% in the ulna). The distal section of the radius presents a thick compact cortex surrounding a small medullary area with a few large cavities separated by thick short trabeculae. It shows very high compactness (about 85%).

Femur

The femur transverse section, slightly distal to the mid-shaft, is very compact (Fig. 6B). A thick layer of compact cortex surrounds a relatively compact medullary area. Because of breakage and of the limited area scanned, it is difficult to determine whether there was a medullary cavity. If present, it must have been much reduced. Compactness is estimated at about 82%.

Tibia

The tibia is also highly compact. A transverse section at about two thirds (distally) of its length shows a very thick and compact cortex and a reduced medullary area with only a few trabeculae, an organization similar to that observed in the distal third of the radius (see above). Compactness is estimated at about 88%. A quite proximal section shows a compact cortical layer surrounding a spongiosa. Compactness remains relatively high (66%).

### (d) Qaisracetus

Rib

The proximal rib fragment of *Qaisracetus* shows a microanatomical organization similar to that observed in the *Rodhocetus* rib.

Vertebrae

Vertebral microanatomical features are again similar to those observed in the *Rodhocetus* vertebra.

Humerus

**Fig 7:**
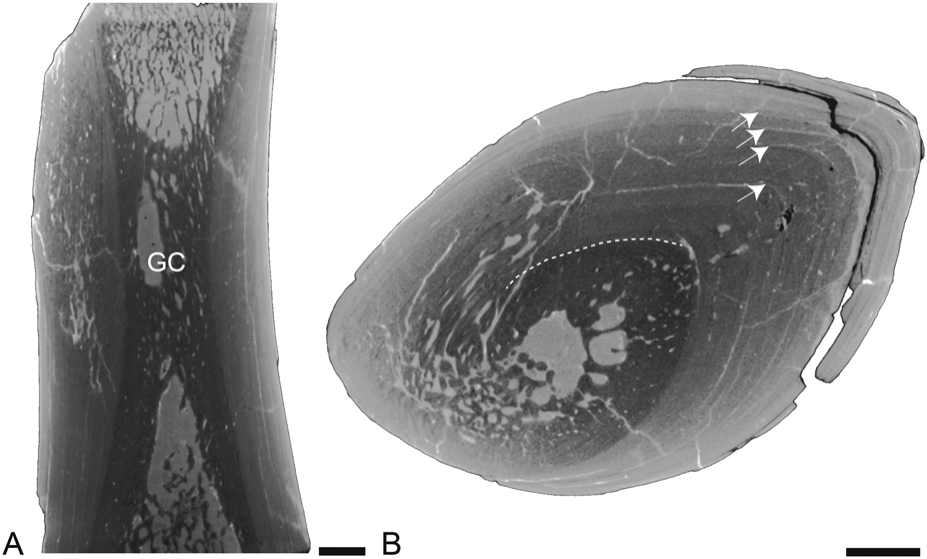
Left humerus of *Qaisracetus arif*. GSP-UM 3318 in virtual longitudinal (A) and transverse (B) sections. The longitudinal section is in posterior view. Scale bars equal 5 mm. Arrows point to LAGs. GC: growth center.

Only the distal half of a humerus is available. The mid-diaphysis is nevertheless well preserved. In longitudinal section, important variations in micro-organization occur along the shaft (Fig. 7A), as in the femur of *Rodhocetus* (see above). Around the center of growth, there is a small and off-center open medullary cavity in a spongious rather small medullary area that is surrounded by a thick layer of compact bone (Fig. 7B). Differences in grey levels (see Fig. 7) seem to indicate the transition between primary periosteal bone (light grey) and secondary bone of both periosteal and endochondral origin (dark grey), as suggested by the observation of LAGs in the light grey area. Periosteal bone appears thus only slightly remodelled. Compactness is very high around the growth center (CI = 91.7 and 92.5) and remains high at some distance from this point. However, it then strongly decreases proximally and distally towards the metaphyses because of thinning of the compact cortical layer and also transformation of the medullary area from more compacted to looser spongiosa (see Fig. 7A).

### (e) Dorudon

Rib

**Fig 8:**
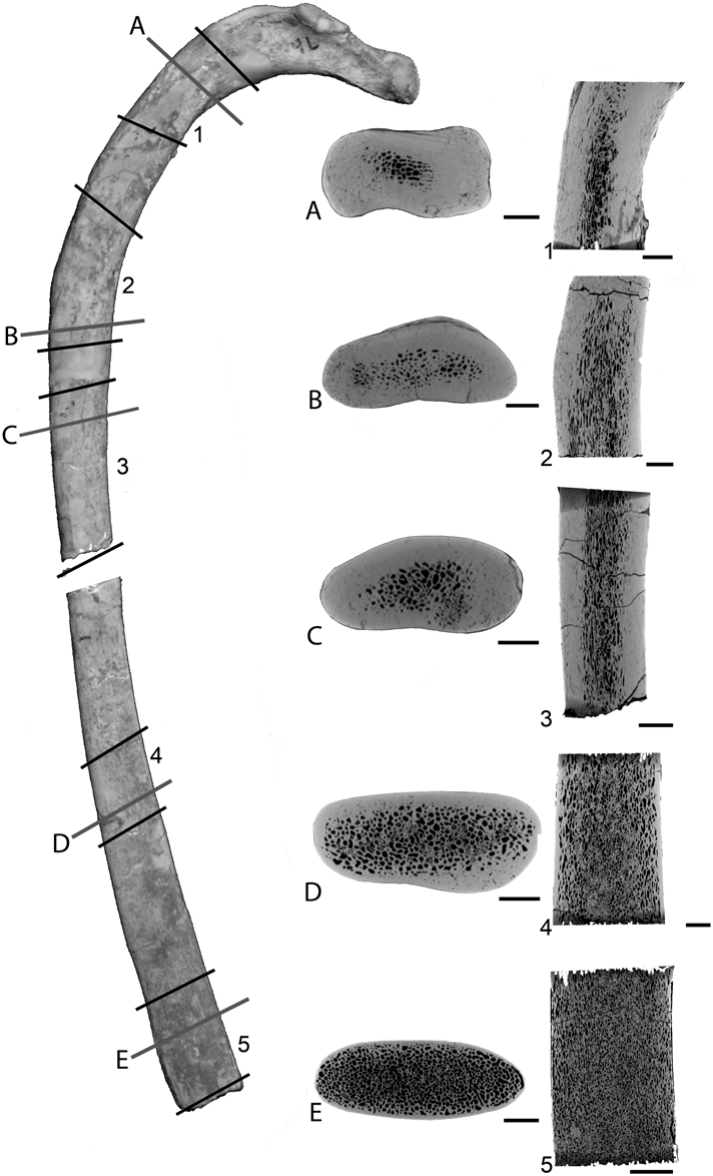
Left rib 4 of *Dorudon atrox* UM 101222 (WH-224). A photo of the rib with scanned segments (1 to 5) and positions of the transverse sections (A to E) labelled is shown on the left in posterior view. Corresponding virtual transverse and longitudinal sections are shown on the right (in center and right columns, respectively). Scale bars equal: A-E, 5 mm; 1–5, 10 mm.

There is a significant variation in microanatomical organization along the rib (Fig. 8). One constant feature is the absence of an open medullary cavity; instead, the medullary area is spongious. The most proximal part of the rib is very compact (CI = 96.1; Fig. 8A), with a thick compact cortex surrounding a rather small spongious medullary area. The latter increases proportionally in size distally. Compactness remains high at about one-third (CI = 91.1; Fig. 8B), one-half (CI = 85.8; Fig. 8C), and two-thirds (CI = 79.2; Fig. 8D) of rib length, but compactness decreases progressively distally while the spongious area becomes the widest. The most distal part of the rib is conspicuously more spongious (CI = 61.3; Fig. 8E) and displays a much thinner compact cortical layer.

Vertebra

The vertebra of *Dorudon* is made of a tight spongiosa. Endochondral and periosteal territories are distinct in longitudinal section; the spongiosa is much tighter in the former, with very numerous thin trabeculae and reduced intertrabecular spaces. There is no surrounding compact layer of periosteal bone.

Humerus

**Fig 9:**
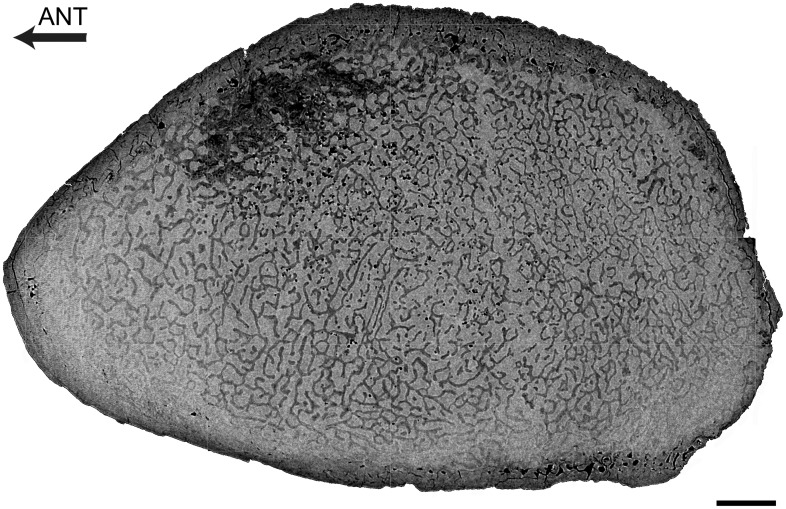
Proximal portion of the left humerus of *Dorudon atrox* UM 101222 (WH-224) in virtual transverse section. Scale bar equals 5mm.

The mid-diaphyseal section of the humerus is almost exclusively a relatively loose spongiosa, with only a very thin layer of compact cortex (Fig. 9).

Radius and ulna

**Fig 10:**
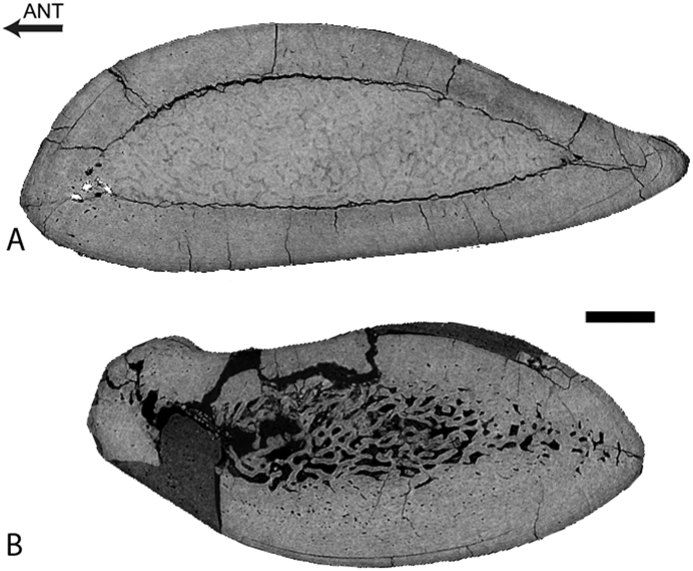
*Dorudon atrox* UM 101222 (WH-224). Virtual transverse sections of the left ulna (A) and radius (B). Scale bar equals 5 mm.

These two bones have, at mid-diaphysis, a thick cortical layer surrounding an entirely spongious medullary area (Fig. 10). Both areas are very clearly distinct (Fig. 10). The spongiosa is loose with rather large trabeculae and intertrabecular spaces. The compactness index of the ulna is difficult to estimate because of the weak contrast between osseous trabeculae and sediment filling intertrabecular spaces (Fig. 10A). The cortex is slightly thicker in the radius (Fig. 10B), making it more compact (CI~82).

Femur

**Fig 11:**
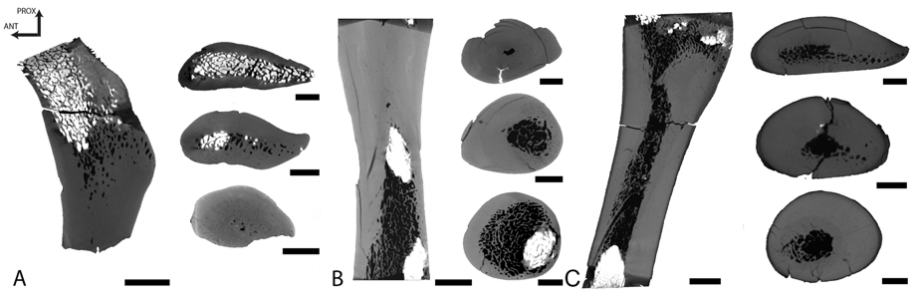
Basilosaurid femora virtual sections. A, Proximal right femur of *Dorudon atrox* UM 97506 (WH-072), longitudinal section in lateral view. B, Distal left femur of female *Basilosaurus isis* UM 97527 (WH-152), in medial view; C, Proximal left femur of male *Basilosaurus isis* UM 93231 (WH-132), in medial view. For all sections, anterior is at the left and proximal at the top. In each panel there is a longitudinal partial section on the left and three transverse sections. Scale bars equal 10 mm for longitudinal and 5 mm for transverse sections.

The femur of *Dorudon* is incomplete. Only the proximal shaft is preserved. The most distal part of the fragment, the mid-diaphysis, is extremely compact (CI = 98.8) and consists only of compact bone with a few small cavities in the core of the section (Fig. 11A). Compactness decreases proximally. In the metaphysis, a loose spongiosa occupies half of the section and is surrounded by a rather thick compact cortex (CI = 64.2).

### (f) Cynthiacetus

Vertebra

The *Cynthiacetus* vertebra is similar to that of *Dorudon*, i.e., spongious with a tight network of numerous thin trabeculae and reduced intertrabecular spaces, notably in the endochondral territory. There is no compact layer of cortical bone in the bone periphery.

Humerus

**Fig 12:**
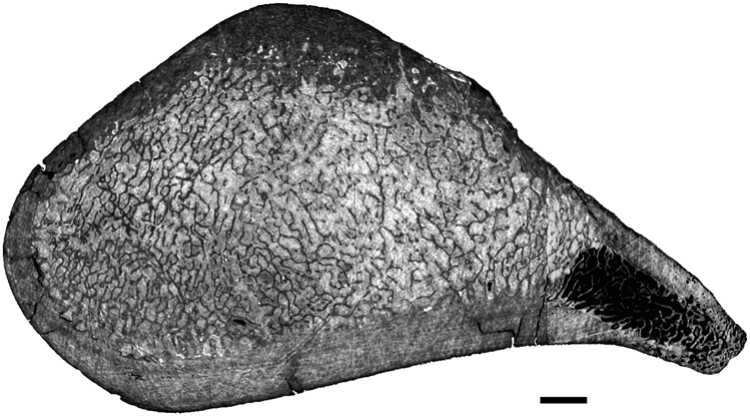
Virtual transverse section of the humerus of *Cynthiacetus peruvianus* MNHN.F.PRU 10. Scale bar equals 5mm.

The mid-diaphysis of the humerus of *Cynthiacetus* is mainly a loose spongiosa (Fig. 12). However, the thin peripheral layer of compact bone is thicker than in *Dorudon*.

### (g) Basilosaurus

Rib

**Fig 13:**
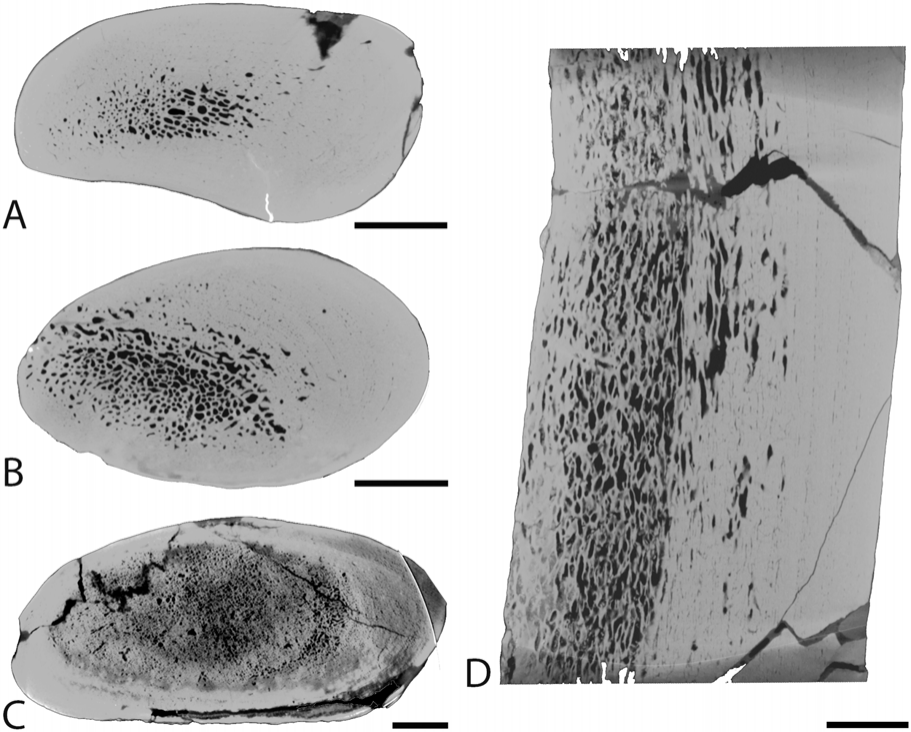
Left rib 4 of *Basilosaurus isis* WH-074. A-C, transverse sections from the proximal, middle, and distal portions of the rib; medial is at the left and posterior at the top. D, longitudinal section from the middle of the rib, in anterior view; medial is at the left and posterior at the top. Scale bars equal 1 cm.

The proximal third of the rib is strongly compact (CI = 95.2). The transverse section shows a very compact cortex with a limited spongious medullary area (Fig. 13A). The latter increases in size distally (Fig. 13B–C)). Compactness remains high at midshaft (CI = 87.9; Fig. 13B). Here the medullary area is strongly off-center, which is evident in both transverse and longitudinal sections (Fig. 13B, D). In the most distal part of the rib, the spongiosa occupies most of the section but is dense and surrounded by a thick layer (especially laterally) of compact cortical bone (Fig. 13C), so that the rib remains strongly compact (CI = 84.9). LAGs are observed in the compact cortex, where remodelling is thus probably limited.

Vertebrae

**Fig 14:**
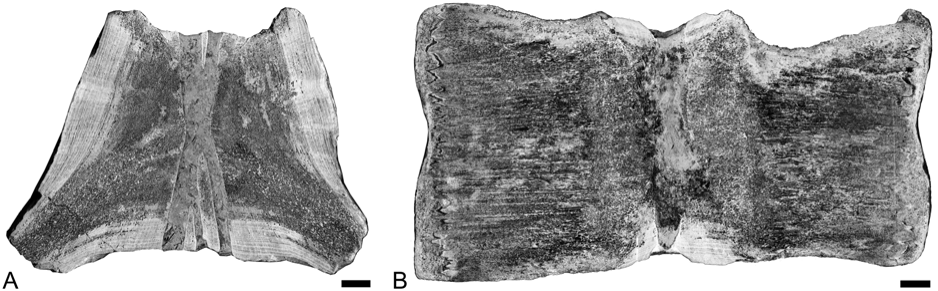
Scanned polished sections of lumbar or anterior caudal vertebrae of *Basilosaurus cetoides*. A- USNM 510831a, transverse section figured in Fordyce & Watson, 1998; dorsal is at the top. B, USNM 510831b, longitudinal section. Scale bars equal 2 cm.

The vertebrae *of Basilosaurus* are spongious and rather similar to that of *Cynthiacetus*, except that thick layers of compact cortical bone, with successive cycles of deposition, are visible along the dorsal and ventral borders of the centrum at its core (Fig. 14A), and along the dorsal and ventral borders of the centrum anterior and posterior to the core (Fig. 14B).

Humerus

**Fig 15:**
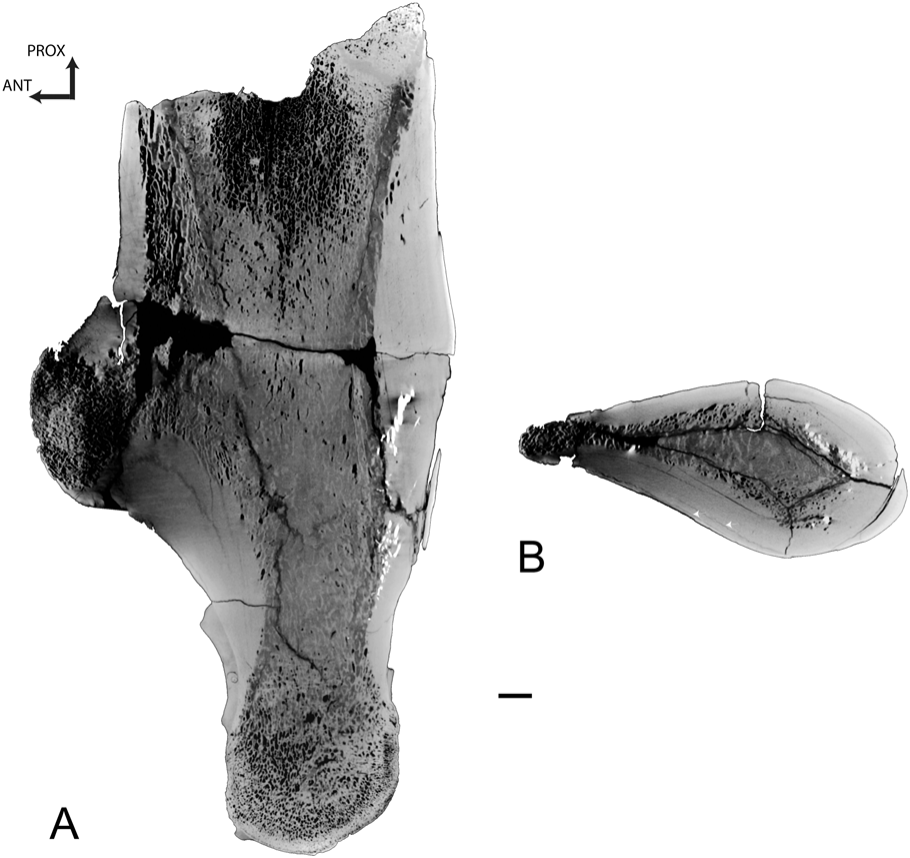
Left humerus of *Basilosaurus isis* UM 94803 (WH-9). A, Longitudinal section of the specimen in lateral view. B, transverse section. Arrows point to LAGs. Scale bar equals 10 mm.

A longitudinal section of the humerus of *Basilosaurus isis* shows that the center of growth is clearly distal in this taxon, being located near the distal end of the deltopectoral crest (Fig. 15A). The humerus shows a thick layer of compact cortical bone that surrounds a spongious medullary area (Fig. 15B). LAGs are observed in this compact bone that probably represents primary periosteal bone. Around the center of growth, the spongiosa is rather open, but its tightness increases proximally and distally (i.e., intertrabecular spaces become smaller and trabeculae thinner).

Femora

Two femora of *Basilosaurus* were analyzed. Both show a strongly compact mid-diaphysis (CI = 98.9 and 90.4 for UM 97527 and UM 93231, a female and a male femur, respectively, see [57]) with an off-center medullary area (Fig. 11B-C), corresponding to an open medullary cavity in the larger male specimen. The center of growth seems located almost at mid-diaphysis, slightly proximally. Compactness decreases away from the growth center, both distally (Fig. 11B) and proximally (Fig 11C). The smaller female specimen (Fig. 11B) shows a rather long part of the diaphysis to be extremely compact, whereas this compactness is more reduced in the male one (Fig. 11C). However, the spongious medullary area away from the compact area is much greater in diameter in the female specimen and the metaphyses are thus much more spongious (see the loose spongiosa in the most distal section of the female specimen in Fig. 11B).

## Discussion

### (a) Bone microanatomical features

Ribs

All ribs lack an open medullary cavity and have unusually high compactness, as compared to other amniotes (see [58]; Table 2). If the *Remingtonocetus* rib is reminiscent of *Enhydra lutris* (see [59]), the other archaeocetes analyzed show a thicker compact cortex and smaller inner cavities. Compactness increases from remingtonocetids to protocetids and from protocetids to basilosaurids and the contrast between a thick compact cortex and an inner spongiosa is much sharper in basilosaurids than it is in protocetids.

**Table 2:** Summary of the microanatomical features observed.

	Remingtonocetus	Rodhocetus	Maiacetus	Qaisracetus	Dorudon	Cynthiacetus	Basilosaurus
**Rib**	No OMC—Spongious organization						
	CIp~80. CId~67	Highly compact. 89.4< CIp/m <91.8. CId = 73.9	X	Highly compact. CIp high	Highly compact. CIp = 96.1,91.1, 85.8. CIm = 79.2. CId = 61.3.	X	Highly compact CIp = 95.2. CIm = 87.9. CId = 84.9
**Vertebra**	Spongious. Layer of compact cortex surrounding the bone periphery and the neural canal	Spongious	Spongious	Spongious	Tight spongiosa	Tight spongiosa	Tight spongiosa. Thick layer of compact cortex surrounding all the centrum around its core
**Humerus**	X	X	Medullary area entirely spongious. Thick compact cortex. CIm = 69	Small off-center OMC Spongious small medullary area Thick compact cortex. CId~92	Relatively loose spongiosa Very thin compact cortex	Loose spongiosa. Very thin compact cortex.	Spongious medullary area. Thick compact cortex
**Femur**	Thick cortex Rather compact inner part Extremely compact in periphery. Off-center OMC. CId = 82	Compact. Off-center OMC. CIp = 83.6 CIm = 87.4. Distally becomes quickly spongious	Very compact. OMC? CIm = 82	X	Very compact. CIp = 98.8	X	Very compact. Off-center medullary areaCI = 98.9 & 90.4.

OMC: open medullary cavity; CI: compactness index (CIp: proximal, CIm; mid-diaphysis, CId: distal).

The proximal halves of *Basilosaurus* and *Dorudon* ribs are notably compact, with compactness indices for the most proximal parts close to those observed in some desmostylians (*Ashoroa*, *Paleoparadoxia* and *Behemotops*; see [56]) and in the semi-aquatic sloth *Thalassocnus* [60]. The microanatomical organization is also generally similar to that of these taxa: a thick layer of compact bone surrounding a reduced spongious medullary area, whereas such an area is not distinguishable in sirenians that show even stronger compactness [59].

There is important change in bone microanatomy along the ribs. The proximal part of the rib is usually the most compact part, with a particularly thick layer of cortical bone. Compactness remains important as far as the midshaft and then decreases distally, the distal portion of the rib usually consisting only of spongiosa. This is however not the case in *Basilosaurus*, where even the distalmost portion of the rib shows a thick layer of compact cortex. This thickening was interpreted as resulting from pachyostosis (see references in [12]). As in sirenians displaying pachyostosis, *Basilosaurus* ribs show a clearly off-center medullary area, the lateral part of the rib growing faster, which might thus be associated with this osseous specialization being more intense laterally.

The marked change in bone microanatomy along the shaft makes homologous comparisons of ribs difficult because there are fewer landmarks to define a reference plane than, for example, in long bones. *Dorudon*’s rib for example resembles ribs of different taxa depending on the region analyzed. From the most proximal region to the most distal one, the rib of *Dorudon* evokes 1) the desmostylians *Paleoparadoxia* and *Ashoroa*, and the nothrotheriid sloths *Thalassocnus littoralis* and *T*. *carolomartini*, 2) the desmostylian *Behemotops*, 3) a modern dolphin, 4) the rorqual *Balaenoptera*, 5) the sirenian *Pezosiren* (see [58,59]). Comparisons must thus be made very cautiously.

It can nevertheless be observed that these archaeocete ribs are all less compact than those of sirenians (except for non-osteosclerotic sirenians, see [59]). However, the archaeocete ribs analyzed all display a clear increase in compactness when compared to extant terrestrial amniotes (see [58]). Similarities, notably for basilosaurid ribs, are observed with the desmostylians *Behemotops*, *Palaeoparadoxis* and *Ashoroa* (see [58]).

Pachyostosis has been mentioned for various archaeocete ribs [12,14]. However, it is neither described nor illustrated in Gray et al. [15], who rely on observation of a thick compact layer of primary periosteal bone in sections without evidence of clear morphological thickening of the bone. The *Zyghoriza* and *Basilosaurus* ribs illustrated in Buffrénil et al. [12] and the *Dorudon* and *Basilosaurus* ribs studied here show some bulging in their distal halves in anterior ribs bound to sternebrae. The thickening evokes what is observed in the desmostylian *Ashoroa* [56] or the youngest species of aquatic sloths, *Thalassocnus littoralis* and *T*. *yaucensis* [60] but it is not comparable to the strong thickening observed in pachyosteosclerotic sirenians. A quantitative anatomical study would be required to clearly determine if this is a common anatomical feature within cetaceans or if it really corresponds to pachyostosis.

The thick peripheral layer of compact cortical bone observed along *Basilosaurus* rib and the osseous drift would be in accordance with the occurrence of pachyostosis (and not only osteosclerosis) with a clear increase in intensity laterally (and not medially as suggested by Buffrénil et al. [12]). Asymmetrical cortical growth also occurs in the pachyostotic ribs of the manatee with also much thicker deposits on the lateral side (see [12]), consistently with the rib morphology and the maintenance of rib curvature during growth. In our sample, if only *Basilosaurus* might display pachyostosis, all other archaeocetes analyzed show only osteosclerosis.

By comparison, *Ichthyolestes* (Pakicetidae) ribs show a tubular structure with a medullary cavity that is clearly open [14], although relatively small (as compared to other amniotes). The cortex is extremely compact and thick. *Pakicetu*s and *Ambulocetus* also display compact ribs with an extremely compact cortex and dense trabecular struts; unfortunately no large scale images of the sections are available so that the occurrence and size of an open medullary cavity remain unclear [14]. *Kutchicetus* (Remingtonocetidae) ribs are strongly compact. They appear more similar to those of the protocetids here sampled than to that of *Remingtonocetus* ([14]; see above). Resorption seems not to have occurred in the outer cortex and remodelling in the inner cortex, and medullary area appears characterized by excessive secondary bone deposits [14], which thus confers on the bone an extremely high compactness. Among Protocetidae, *Gaviacetus* ribs are also very dense, but *Georgiacetus* ones are less compact and show more numerous thinner struts [14]. *Zygorhiza* ribs show a wide cancellous medullary area [12,14]. Further investigations would be required to determine the degree of osteosclerosis in *Zygorhiza*.

Vertebrae

All archaeocete vertebrae analyzed are spongious (Table 2). The variations observed in archaeocete vertebrae, except for *Remingtonocetus* and *Basilosaurus* (see below), are variations in tightness of the spongiosa (i.e. of the trabecular network), which increases with specimen size (trabeculae becoming more numerous and thinner with smaller intertrabecular spaces). This positive (qualitative) correlation was already quantitatively highlighted in various amniotes [49,61]. *Remingtonocetus* displays a circumferential layer of compact cortex, like the extant polar bear but not to the extent of the hippopotamus and manatee (see [58]). It evokes a condition intermediate between those of the desmostylians *Behemotops* and *Ashoroa*, respectively (see [58]). Comparisons with diverse amniotes (cf. [58,61,62]) show that the other (more derived) archaeocetes have a vertebral micro-organization similar to that of modern cetaceans. Only *Basilosaurus* differs from this condition, with a thick layer of compact cortical bone surrounding the middle of the centrum (the centrum being the only part of the vertebra available for this study) but also the neural arch and transverse processes (PDG. pers. obs.). This layer is thicker than in the extant *Hippopotamus*, *Choeropsis* and *Trichechus* (cf. [58,61]). To our knowledge, such a structure (engendering local bone mass increase [BMI]) has not been observed in any other (extant or fossil) taxon. This peculiarity is, moreover, not associated with any morphologically observable bulging and thus does not correspond to pachyostosis. It thus differs from the condition observable in *Basilotritus* vertebrae, which show laterally swollen neural arches and robust zygapophyses [63]. *Basilosaurus* condition seems related to the very peculiar morphology of its vertebrae and might reflect a structural requirement for these large spongious vertebrae related to muscle insertion and locomotion.

Humeri

The longitudinal sections clearly show an important change in bone microanatomy along the diaphysis. This condition is unusual in amniotes, whatever their ecology. It has so far only been observed in *Enhydra lutris* and in turtles and fossil ichthyosaurs and plesiosaurs [64,65]. For this reason, homologous comparisons require a precisely cut transverse “perfect diaphyseal plane” (sensu [64]), i.e., the plane cutting the point where growth originated (see [65] for more details about this sectional plane). In order to locate such a cut, a longitudinal section of a rather long part of the central diaphysis is required. Unfortunately, because only very short mid-diaphyseal portions were imaged in *Maiacetus*, *Dorudon* and *Cynthiacetus*, perfectly homologous comparisons cannot be made. The longitudinal sections of *Qaisracetus* and *Basilosaurus* show that the center of growth is not located at mid-shaft but much more distally. This suggests that growth was much faster proximally than distally in the humerus in these taxa. Because of the weak remodelling of compact cortical bone (as suggested by the grey-level differences reflecting density differences, and, especially, by the observation of LAGs), the area around the center of growth is the more compact one. The spongiosa is extended much farther away from the center of growth.

The transverse section of the humerus of *Qaisracetus* evokes the condition observed in some fossil marine sauropterygians (e.g. *Cymatosaurus*, *Anarosaurus*, *Placodus*; [66]). It is the only humerus analyzed in which a medullary cavity, though very small, is observed (Table 2). The section of *Maiacetus*, from the mid-diaphysis, evokes what is observed in some otariids and placodonts (AH, pers. obs.); however this section is probably at some distance from the perfect diaphyseal plane and it cannot be determined whether a medullary cavity was present around the centre of growth. Distance from the diaphyseal plane would also explain the relatively large spongious medullary area in the *Maiacetus* section. However, even the *Maiacetus* section clearly shows an increase in bone compactness as compared to extant amniotes, with a thick layer of compact cortex and a spongious medullary area but lacking an open medullary cavity.

The humerus of *Basilosaurus* shows a thick cortex. However, it is distinctly thinner in *Cynthiacetus* and even more reduced in *Dorudon*, whose section is almost entirely spongious. The sections of these last two taxa resemble the condition in modern cetaceans, with the layer of compact cortex in *Cynthiacetus* being thinner than in *Platanista*, and that of *Dorudon* being similar to those of *Delphinus* or *Lagenorhynchus* (AH, pers. obs.; [67]). Conversely, the proportional thickness of compact cortex of *Basilosaurus* sections evokes what is observed in *Enhydra*, *Lutra* or *Leptonychotes*. However, the medullary area of *Basilosaurus* is spongious, whereas it is almost open with only a few trabeculae in these taxa.

The humerus of *Ichthyolestes* (Pakicetidae) was studied by Thewissen et al. [68]. It shows an extremely compact tubular structure with a very thick compact cortex and a reduced (also off-centered) open medullary cavity, and thus appears rather similar to the *Qaisracetus* transverse section described above.

Femora

*Remingtonocetus* and *Rodhocetus* femora show a similar microanatomical organization (Table 2), although the inner cortex appears more compact in *Rodhocetus*. The variation in inner bone structure along the diaphysis is rather similar to what was described in the humerus but the growth center is located proximally in the femur. Unfortunately it cannot be determined whether the *Maiacetus* transverse section is close to the growth center. Moreover, because of breakage, it is difficult to determine whether a medullary cavity was present. If it was, it would have been smaller than those in *Remingtonocetus* and *Rodhocetus*. The bone was probably much more compact.

Basilosaurid femora clearly differ from the others (Table 2). *Dorudon*’s femur is strongly compact with no medullary cavity. Away from the metaphysis, the diaphysis is extremely compact. *Basilosaurus* femora are also strongly compact. The growth center appears also rather proximal in these taxa. The differences between the two *Basilosaurus* specimens studied here are associated with an important bone size difference. This variation does not result from ontogeny, as the smaller specimen is clearly not a juvenile from an anatomical perspective and as suggested by the multiple possible growth marks observable on the sections. It could rather result from sexual dimorphism, as suggested by Gingerich et al. [69] and Antar et al. [56].

*Remingtonocetus* and *Rodhocetus* femoral sections resemble those of some fossil marine reptiles (e.g., *Nothosaurus*, *Simosaurus*) and of the modern *Alligator* and *Trichechus manatus* (manatee). Basilosaurid femora are more compact, to our knowledge, than in any extant amniote. It evokes very compact femora of some fossil sauropterygians (e.g., *Paraplacodus*, *Pistosaurus*; [70]).

Femora of *Ambulocetus*, *Rodhocetus*, *Remingtonocetus* and *Basilosaurus* were analyzed by Madar [13], based on radiographs, in order to document the distribution of compact and spongious bone and the possible occurrence of an open medullary cavity. Our observations for the femora of *Remingtonocetus* and *Rodhocetus*, based on the same specimens, differ substantially. Madar [13] found the cortical bone in *Remingtonocetus* to be extremely thin near mid-shaft. The compact cortex is indeed thin but not as extremely as indicated by Madar. If Madar did not note the occurrence of an open medullary cavity, she nevertheless observed a difference in compactness in the medullary area (see [13]: Fig. 4) corresponding to the contrast between the inner cortex (a very compact spongiosa) and the medullary cavity. Madar [13] described a thin cortex all along the diaphysis in *Rodhocetus* and did not observe any medullary cavity. Dense mineralization of *Rodhocetus* (cf. [13]) undoubtedly affected her radiographs.

Madar’s observations on *Basilosaurus* are more consistent with our results. However, contrary to what Madar [13] suggested, *Basilosaurus* femora do display an open medullary cavity, although it is very small around the growth center. Cortical bone deposits are extremely compact along the whole diaphysis. Such an osteosclerotic pattern is similar to that observed in some desmostylian (*Ashoroa*, *Paleoparadoxia* and *Behemotops*), the aquatic sloth *Thalassocnus carolomartini* [60] and sirenian long bones [58,67]. However, no longitudinal section is available for these taxa, so that the variations along the diaphysis observed in *Basilosaurus* cannot be compared. They are however clearly distinct from the pattern usually observed in amniote long bones ([71]; A.H. pers. obs.), which have a tubular diaphysis with a rather homogeneous thickness of compact cortical bone all along the diaphysis.

Zeugopod bones

Bones of the zeugopodium (ulna-radius and/or tibia-fibula) were only analyzed for *Maiacetus* and *Dorudon* and, unfortunately on a very short section of the diaphysis. Thus, it is unknown whether the microanatomical differences observed in the archaeocete stylopodium (humerus and/or femur) also occur in the zeugopod. However, the distal section of the *Maiacetus* radius shows a more compact structure than the mid-diaphyseal section. This suggests a rather distal location of the growth center and a high compactness around this point as in stylopod bones. Sections of *Dorudon* suggest greater compactness in stylopod bones, with a thicker layer of compact cortex. However, longitudinal sections will be required to confirm this.

### (b) Swimming behaviour

Remingtonocetus

*Remingtonocetus* rib is spongious. The femur displays a tubular structure but the open medullary cavity is rather small. These two bones generally show high compactness values, as compared to other amniotes (see [58]). The microanatomy of the rib, vertebra and femur evokes that of the sea otter, polar bear, and of some not actively swimming semi-aquatic to aquatic reptiles (see above). These microanatomical features are thus in accordance with anatomical and geological data to assume an amphibious, though essentially aquatic, lifestyle. The compact femur indeed suggests a difficult hind-limbs-supported terrestrial locomotion. Indeed, such a thick compact cortex is observed either in semi-aquatic taxa with a rather poorly active terrestrial locomotion or in graviportal ones (A.H. pers. obs.). Based on its morphological and microanatomical features, *Remingtonocetus* is thus assumed to have displayed extremely limited terrestrial locomotion. Bebej et al. [19] highlighted a lack of mobility between functional series of vertebrae, as opposed to the condition observed in protocetids and basilosaurids. In accordance with this result, adaptations to an aquatic life at the microanatomical level appear more limited as compared to these other archaeocetes (see below). However, Bebej et al. [19] considered the rest of the morphology as indicating active foot-powered swimming, which appears only slightly compatible with the occurrence of BMI, based on our current knowledge. The recent suggestion of this mode of swimming in the semiaquatic dinosaur *Spinosaurus aegyptiacus* displaying strong BMI in its hindlimbs [72] would nevertheless agree with the previous hypothesis. Further comparisons with extant semi-aquatic taxa would be required to see if active foot-powered swimming can be consistent with the microanatomical features of *Remingtonocetus* and to propose more precise inferences about its possible swimming style.

Protocetids

*Rodhocetus* microanatomical features are very similar to those of *Remingtonocetus*. However, the vertebra does not display compact layers surrounding the neural canal and the bone periphery. Moreover, the rib and the femur are more compact. This increase in bone mass suggests a stronger need for buoyancy control in *Rodhocetus* than in *Remingtonocetus* and an even less efficient terrestrial locomotion in *Rodhocetus*. *Rodhocetus* would thus have been more adapted for underwater swimming, probably slowly and at shallow depth (see [45]), than *Remingtonocetus*. The data concerning *Maiacetus* long bones are not as accurate as those from *Rodhocetus* but seem rather similar. *Qaisracetus* data are also consistent with what is observed in the other protocetids. The very small medullary cavity observed in the humerus shows a high degree of BMI. These protocetids, with their compact long bones and ribs, probably had considerable difficulties withlocomotion on land. However, contrary to some previous assumptions (see introduction), the occurrence of BMI in the long bones and ribs of the protocetids sampled is more compatible with suspended swimming in shallow waters than with a pelagic life.

Basilosaurids

*Dorudon* shows a spongious, rather lightly built humerus but compact ribs, in at least their proximal half, and a strongly compact femur. Femora in *Dorudon* are greatly reduced bones not involved in locomotion. A similar BMI is observed in *Basilosaurus* femora. Such regressed limb elements with a supposedly similar function also occurred in Late Cretaceous hind-limbed snakes. However, if the latter display BMI in much of their skeleton [46], their femora are deprived of this osseous specialization [73]. The occurrence of BMI in regressed hind-limbs remains unexplained. Despite the femur microanatomy, the other bones of *Dorudon* analyzed show microanatomical features very similar to those of modern dolphins. This is not the case for *Basilosaurus* whose ribs show a higher inner compactness (osteosclerosis) and what seems to correspond to increased periosteal bone deposits (pachyostosis). Only a humerus and a vertebra of *Cynthiacetus* were analyzed. Both bones show a microanatomy more similar to *Dorudon* than to *Basilosaurus*. *Basilosaurus* thus appears as peculiar among basilosaurids. In addition to its peculiarly long vertebrae characterized by the occurrence of a yoke of compact bone surrounding the mid-centrum, neural arches and transverse processes, *Basilosaurus* also displays 1) probable pachyostosis in its ribs and 2) osteosclerosis in its humerus. The occurrence of pachyostosis in the ribs was also documented in *Zygorhiza* and the expanded distal extremities of ribs 3 to 7 of *Cynthiacetus peruvianus* (CdM; pers. obs.) also suggests pachyostosis in this taxon. However, rib general morphology and osseous microstructure need to be further investigated in basilosaurids, and more generally in cetaceans in order to clearly determine whether pachyostosis really occurs in *Basilosaurus*, *Zygorhiza*, and *Cynthiacetus*. Moreover, rib microanatomical features, and notably the variation along the bone, need to be further investigated in *Zygorhiza* and no data are available concerning other bones. The occurrence of BMI in *Basilosaurus* is surprising as this taxon is generally considered an active predator. BMI was assumed to be associated with its particularly long (serpentine) post-thoracic region to assist in body trim control [12]. However, this argument cannot be used for *Zygorhiza* and *Cynthiacetus*, which show a length of the post-thoracic region similar to those of modern mysticetes and odontocetes. Moreover, body trim control is not compatible with BMI in lumbar, and thus rather posterior, vertebrae. The occurrence of this specialization, at various degrees of intensity, in several bones of this taxon remains mysterious. Further comparisons among basilosaurids and with large modern whales are required to better understand its significance.

Fordyce and Watson [74] described some “archaic fossil mysticeti” from New Zealand as showing osteosclerosis or “peripheral osteosclerosis”, after describing the vertebra USNM 510831a (Fig. 14) as itself osteosclerotic. Further investigations and comparisons would be required in order to determine whether thespecialization in *Basilosaurus* resembles that of these early mysticetes.

## Conclusions

Analysis of the microanatomical features of various bones of three of the five archaeocete families enables us to discuss evolutionary trends in the progressive adaptation to an exclusively aquatic life in the cetacean lineage, and to make paleoecological inferences for the taxa studied.

1. Ribs of the Remingtonocetidae, Protocetidae and Basilosauridae sampled here lack an open medullary cavity. All these taxa display bone mass increase (BMI) in their ribs and femora, while in contrast having essentially or exclusively spongious vertebrae. In the protocetids studied humeri and femora are essentially compact with a small open medullary cavity around the growth center. In this respect, protocetid humeri resemble humeri of the pakicetid *Ichthyolestes*, but they differ markedly from the essentially spongious humeri of basilosaurids. As opposed to Remingtonocetidae and Protocetidae, basilosaurids display very compact femora. Anterior and posterior long bones thus show clearly distinct trends in microanatomical specialization in the progressive independence from a terrestrial environment, which is naturally associated with the functional role of these bones. Forelimbs progressively lost any propulsive role and became used for steering and stabilization, consistent with acquisition of a spongious organization, whereas hind limbs became strongly reduced and lost any involvement in locomotion. The occurrence of strong osteosclerosis in these reduced appendages remains unexplained.
2. Our observations are in accordance with previous geological and anatomical data that suggest an amphibious lifestyle with very limited terrestrial locomotion for both the remingtonocetids and the protocetids sampled. Basilosaurids, on the other hand, show specializations similar to modern cetaceans and were clearly more actively swimming in the open sea. *Basilosaurus* itself is unusual among basilosaurids in displaying bone mass increase in its ribs and long bones, although with various intensities, with a yoek of compact bone surrounding the mid-centrum, neural arches, and transverse processes of most vertebrae, which are unusually long. The observation of BMI in posteriorly-located bones shows that this specialization occurs for reasons other than body trim control. BMI in posteriorly located bones is poorly compatible with *Basilosaurus* morphology in general and with its presumed behavior and ecology, and BMI in *Basilosaurus* remains to be explained.
3. This study also highlights the significant variation in bone microanatomy observable along the shaft of the ribs and the diaphysis of long bones, showing that comparisons have to be made with caution in order to deal with homologous regions.
4. The previous works by Madar [13] and Gray et al. [14] were the most substantial contributions available previously on archaeocete bone microanatomical features. Both studies covered the five archaeocete families. However, they focused on a single bone (the femur and the rib, respectively). It is important to combine information from various bones to get a better idea of the variation in degrees of adaptation to aquatic life during the land to sea transition.
